# Cotton soot derived carbon nanoparticles for NiO supported processing temperature tuned ambient perovskite solar cells

**DOI:** 10.1038/s41598-021-02796-w

**Published:** 2021-12-03

**Authors:** Shubhranshu Bhandari, Anurag Roy, Mir Sahidul Ali, Tapas Kumar Mallick, Senthilarasu Sundaram

**Affiliations:** 1 Environment and Sustainability Institute (ESI), Penryn Campus, University of Exeter, Cornwall, TR10 9FE UK; 2 Department of Polymer Science and Technology, University of Calcutta, 92 A.P.C Road, Kolkata, 700009 West Bengal India

**Keywords:** Energy, Materials chemistry

## Abstract

The emergence of perovskite solar cells (PSCs) in a "catfish effect" of other conventional photovoltaic technologies with the massive growth of high-power conversion efficiency (PCE) has given a new direction to the entire solar energy field. Replacing traditional metal-based electrodes with carbon-based materials is one of the front-runners among many other investigations in this field due to its cost-effective processability and high stability. Carbon-based perovskite solar cells (c-PSCs) have shown great potential for the development of large scale photovoltaics. First of its kind, here we introduce a facile and cost-effective large scale carbon nanoparticles (CNPs) synthesis from mustard oil assisted cotton combustion for utilization in the mesoporous carbon-based perovskite solar cell (PSC). Also, we instigate two different directions of utilizing the carbon nanoparticles for a composite high temperature processed electrode (HTCN) and a low temperature processed electrode (LTCN) with detailed performance comparison. NiO/CNP composite thin film was used in high temperature processed electrodes, and for low temperature processed electrodes, separate NiO and CNP layers were deposited. The HTCN devices with the cell structure FTO/c-TiO_2_/m-TiO_2_/m-ZrO_2_/high-temperature NiO-CNP composite paste/infiltrated MAPI (CH_3_NH_3_PbI_3_) achieved a maximum PCE of 13.2%. In addition, high temperature based carbon devices had remarkable stability of ~ 1000 h (ambient condition), retaining almost 90% of their initial efficiency. In contrast, LTCN devices with configuration FTO/c-TiO_2_/m-TiO_2_/m-ZrO_2_/NiO/MAPI/low-temperature CNP had a PCE limit of 14.2%, maintaining ~ 72% of the initial PCE after 1000 h. Nevertheless, we believe this promising approach and the comparative study between the two different techniques would be highly suitable and adequate for the upcoming cutting-edge experimentations of PSC.

## Introduction

Perovskite solar cells (PSCs) have explored significant breakthroughs in the field of third-generation solar cells and spurred researchers to develop and experiment with a variety of new materials and architectures, achieving > 25% power conversion efficiency (PCE)^1–3^. Due to the easy fabrication methods and higher PCE, PSCs are often used as sources of self-powering systems and portable/wearable electronics^4^. Recently, Huan et al. disclosed an economic photovoltaic-electrochemical system comprising a low-cost perovskite photovoltaic mini-module, manifesting ~ 2.3% solar-to-hydrocarbon efficiency^5^. Massive amount of work is going on for the commercialization of perovskite-based photovoltaic technology as well^6–8^. These cells often consists of mesoporous layers, a simple fabrication process, high energy conversion and enhanced resistivity towards environmental factors^9,10^. The selection of materials and their fabrication process has limited PSCs' performance as highly efficient PSCs often lose their PCE due to the nature of the materials used^11^. Various materials, device structures, and manufacturing techniques are being pursued, and it is unclear which of these approaches is the most promising. Usually, PSC components like electron transport material (ETM), perovskite sensitizer, hole transport material (HTM), and electrode materials need to be appropriately aligned according to the electron transfer and recombination process in order to achieve the best out of the device^12,13^. The highly-rated TiO_2_ is the most promising ETM in the n–i–p structure, although SnO_2_, ZnO, doped BaSnO_3_ are not far behind the scene^14–17^. On the other hand, Fullerene and its derivatives are the most utilized ETM for the p–i–n structure^18^. The commonly used organic counterparts of the ETM for p–i–n structure always have issues in making stable and cost-effective PSC, giving an edge to the n–i–p devices^19,20^. Perovskite, being the most important component of PSC devices, needs to cope up with the ETM strongly^21,22^. Numerous research works are occurring spontaneously to develop stable and efficient perovskites, and so far, auspicious results have been achieved. Starting from CH_3_NH_3_PbI_3_ (MAPI), development of mixed iodide, fully inorganic, 2D/3D mixed cationic (methylammonium, formamidinium etc.), and Pb-free perovskites have shown paths towards commercialization^23^. Now, it is up to the HTM to show their effectiveness in producing the PCE of devices comparable to traditional silicon-based devices^24^. Typically 2,2',7,7'-Tetrakis [N,N-di(4-methoxyphenyl)amino]-9,9'- spirobifluorene (Spiro-MeOTAD), graphene/poly(3,4-ethylenedioxythiophene)polystyrene sulfonate (PEDOT:PSS), NiO, CuSCN, Cu_2_O etc. are employed as HTM for n-i-p model^25–27^. Similarly, poly[bis(4-phenyl)(2,4,6-trimethylphenyl)-amine] (PTAA), (PEDOT:PSS), NiO_x_ etc. have been used as the HTM for p-i-n structure^28,29^. Despite the high PCE of organic HTM based devices, they are vulnerable to light and temperature treatment^25,26^. In contrast, inorganic HTM can resolve the stability issues, although they produce less PCE^32^. Efforts are going on to generate more PCE with inorganic HTM based devices by introducing materials like Co_3_O_4_, Fe_3_O_4_, WO_3_ etc^33–35^. The electron transfer process greatly depends on the perfect contact between the HTM and counter electrode^36,37^. Traditional gold, silver and aluminium electrodes are the most efficient ones in terms of PCE, although their large scale cost of production is a matter of concern along with their reactivity with perovskite materials to facilitate decomposition^38^. Standard PSC with carbon-based back contact is a suitable solution to substitute noble metals due to their low cost, high conductivity, eventual low-temperature processing and work function close to gold^39–43^. Various allotropes of carbon as well as composite HTM-based carbon have been extensively used as a counter electrode to facilitate the better performance of devices^18,44–48^.

Here, we report a possible way of synthesizing carbon nanoparticles (CNP) on a large scale from the combustion of cotton fabrics in the presence of mustard oil for utilization in PSC. The collected CNP was used as a low-temperature counter electrode as well as a composite high-temperature counter electrode with NiO (HTM) for different types of perovskite devices. These two different routes can give us the time of production, cost of production and reliability of the entire fabrication processes, and performance behaviour to understand their individual efficacy. For low-temperature devices, the configuration used is as follows FTO/c-TiO_2_/m-TiO_2_/m-ZrO_2_/NiO/MAPI/low-temperature carbon. On the other hand, high-temperature counter electrode-based devices were comprised of the following structure FTO/c-TiO_2_/m-TiO_2_/m-ZrO_2_/high-temperature NiO-CNP composite paste/infiltrated MAPI. Comparison of performance was also carried out between commercial carbon black and the synthesized CNP for a better apprehension of advantages and disadvantages. Observed trends exhibit that the low-temperature electrode-based device with CNP can achieve the best PCE of ~ 14.2% compared to ~ 13.2% PCE of high-temperature-carbon-based devices, although the stability of HTCN (high-temperature carbon nanoparticles) devices is higher than that of the LTCN (low-temperature carbon nanoparticles) devices. This comparative analysis can lead us to understand the favourable process for large scale fabrication of CNP counter electrode perovskite solar cell (c-PSC) towards commercialization.

## Experimental section

### Materials preparation

**Figure 1:**
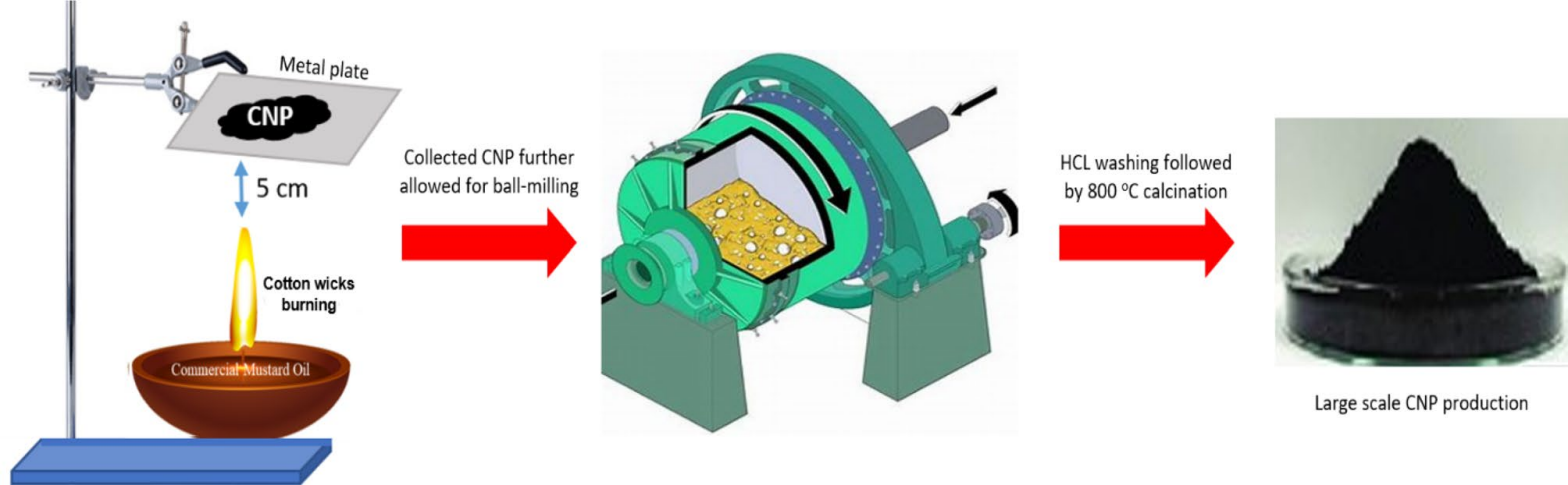
Schematic representation of carbon nanoparticles synthesis utilizing burnt cotton wicks.

Commercial mustard oil bought from a local market in Kolkata, India, was placed in a container (15 ml), and cotton wicks (30 mg) were immersed over the oil (^**^declaration for the usage of plant materials is mentioned after characterizations section). Then the cotton was burned with fire in the presence of the oil, and from this combustion, black powders were collected in a metal plate kept 5 cm apart from the flame. The collected black powder was then transferred to a grinding container for the next step. These powders were then treated with 2 M HCl and neutralized by NaOH after grinding by ball milling. After that, it was annealed at 800 °C to remove unreacted impurities like hydrocarbons, plasma membranes, fatty substances and degrade amorphous carbon, which enhances the conductivity^49,50^. Finally, carbon nanoparticles were collected in a petri dish before further characterization, as shown in Fig. 1. From 30 mg of cotton wicks, 100 mg of carbon nanoparticles were obtained, indicating that large-scale synthesis is highly possible using this method.

Synthesized CNP 1.2 g, 0.1 g graphite and 1.8 g of NiO (< 50 nm, purchased from sigma) were dispersed in ethanol (15 mL) and sonicated for 30 min. The solution was then ultrasonicated for 5 min and stirred for 10 min. Next, 13 mL terpineol was added to the solution. After that, 15 g ethylcellulose was added into the mixture and treated by an ultrasonicator for 5 min, magnetic stirring for 10 min, and ball-milling for 8 h. In this way, HTCN paste with NiO was prepared. For carbon black paste, CNP was replaced by commercial black carbon.

For LTCN paste, 2 gm CNP, 0.25 gm graphite and 15 ml chlorobenzene was ball milled for 8 h. The collected paste was utilized for the blade-coating technique. The use of a small amount of graphite is highly suitable for better contact of the electrode with the perovskite layer^51,52^.

Low-temperature carbon black paste was prepared in the same way to that of the LTCN paste. In this case graphite and carbon black were mixed at a 1:2 ratio in 20 ml of chlorobenzene via ball milling^53^.

The perovskite solution was prepared by dissolving 1.2 M MAI and 1.2 M PbI_2_ in γ-butyrolactone (GBL) solution, followed by stirring overnight at 60°C^54^. The freshly prepared precursor solution was then filtered and utilized for the high-temperature counter electrode (HTCE) based devices.

Again, a mixture of 462 mg of PbI_2_ and 163 mg of CH_3_NH_3_I dissolved in Dimethylformamide (DMF) and dimethyl sulfoxide (DMSO) mixed solvent (v/v = 7:3) under stirring at 70 °C was prepared for the low-temperature counter electrode (LTCE) based devices^55^.

### Device fabrication

The methodology was adopted from earlier reported articles with suitable modifications, as shown in Fig. S3, SI (supporting information)^33,46^. The entire device fabrication process was carried out under ambient conditions for all cases, and the image of as-prepared devices is given in Fig S1a, SI. Fluorine doped tin oxide (FTO) glass substrate (2 cm × 2 cm) was etched in the first step, followed by standard cleaning procedures, which was common for both the high-temperature and low-temperature carbon electrode-based devices. Next, titanium di-isopropoxide bis-(acetylacetonate) (TDBA) (75 wt.% in isopropanol, Sigma-Aldrich) solution in 2-propanol (1:6 v/v) was prepared for deposition in high-temperature carbon-based devices. On the other hand, titanium isopropoxide (TTIP) in HCl and ethanol was used as the precursor solution of the blocking-TiO_2_ layer for low-temperature carbon-based devices. The compact-TiO_2_ layer was spin-coated using the above mentioned two different precursor solutions for the two different devices, at 3000 rpm for 30 s, followed by heating at 415 ± 10 °C for 30 min and cooling to room temperature. In the 3rd step, the mesoporous TiO_2_ layer was deposited by spin coating at 4000 rpm for 30 s using diluted TiO_2_ paste (18NRT from Great cell Solar Company; w/v = 1:6 in ethanol) and heated at 500 °C for 60 min. After cooling to room temperature, 0.1 M solution of Lithium (trifluoromethane sulfonyl) imide (LITFSI) in acetonitrile was spin-coated (3500 rpm for 20 s) on top of the m-TiO_2_ layer followed by annealing at 450 °C. The ZrO_2_ mesoporous layer was spin-coated with diluted ZrO_2_ paste (Solarnix; v/v = 1:5 in ethanol) at 4000 rpm for 30 s and heated at 450 °C for 30 min (step 4). After that, the devices were divided into two sets. For one set, the NiO layer was deposited using nano-oxide based paste (Solarnix, diluted at 1:5 v/v in ethanol) at 4000 rpm 30 s for the low-temperature carbon-based devices and sintered at 450 °C for 30 min (step 5b). After cooling down to room temperature, the MAPI precursor solution with an appropriate amount (50 μl) was spin-coated at 1000 and 5000 rpm for 10 s and 20 s, respectively. During the last 10 s of rotation, chlorobenzene (400 μl) was splashed from the top (step 6b). Finally, the low-temperature carbon electrode was deposited by blade coating and heated at 100 °C for 5 min (step 7b). For another set of devices, high-temperature carbon nanoparticles-NiO composite paste was screen printed above mesoporous ZrO_2_ layer to obtain mesoscopic carbon layer, which was sintered at 400 °C for 30 min (step 5a). Finally, perovskite precursor solution was infiltrated by drop-casting via the top of the counter electrode and further spin-coated at 3000 rpm for 15 s followed by drying at 50 °C for an hour (Step 6a). Device fabrication using the commercial carbon black as the counter electrode was carried out in the same manner as the synthesized CNP based devices. For each case, a set of 10 devices were fabricated, and a couple of images of fabricated devices are given in the supporting information Fig. S1b. At last, the PSCs were employed for further characterization and performance check.

### Characterizations

The room temperature powdered X-ray diffraction (XRD) was performed for synthesized nanoparticles by Philips X-ray diffractometer (PW1730) with Cu Kα radiation at a 2θ scan rate of 2° per minute. Raman spectroscopy of CNPs was carried out using Renishaw Reflex micro-Raman spectrometer with an argon ion, 514 nm laser. A Tecnai G2 30ST (FEI) high-resolution transmission electron microscope (TEM) operating at 300 kV was used for transmission electron microscopy (TEM) of CNPs. Thermogravimetric analysis (TGA) was done utilizing Perkin Elmer (PYRIS-1) instrument. The PSC's cross-sectional layer thickness measurement and elemental mapping were recorded on a scanning electron microscope (SEM–EDX) (LEO 430i, Carl Zeiss). XRD study of the fabricated devices was executed on an X'pert pro MPD XRD of PAN analytical with Cu Kα radiation (λ = 1.5406 Å). Further, photovoltaic measurements of the PSC were executed in reverse bias condition under 1000 W/m^2^ of light illumination from a Wacom AAA continuous solar simulator (model type: WXS-210S-20, AM1.5G) and an EKO MP-160i *I–V* Tracer (the instrument performed 256 data points measurement having a sweep time of 5 s). EIS assessment was performed with an AUTOLAB frequency analyzer setup equipped with an AUTOLAB PGSTAT 10, and a Frequency Response Analyzer (FRA) module under the dark condition having a frequency range from 1 MHz to 10 mHz at the 0.80 V open-circuit voltage. The Z-view software (version 3.4d, Scribner Associates, Inc., USA) was used to fit the experimental data. Incident photon to current efficiency (IPCE) measurement was carried out on a BENTHAM PVE300 Photovoltaic EQE and IQE solution under 350–750 nm wavelength using a tungsten halogen lamp source^45^. All the schematic diagram of this work has been created using the Microsoft PowerPoint 2019 MSO (Version 2109 Build 16.0.14430.20224), and Chemdraw Ultra (version 8.0) by Cambridge soft corporation (Website: http://www.cambridgesoft.com/). SEM analysis, HRTEM analysis, SAED crystal plane identification, and d-value calculations were performed using Image J 1.40 g (Wayne Rasband, National Institute of Health, USA, website: http://rsb.info.nih.gov/ij/). The electrochemical impedance experimental data were fitted with the Z-view software (version 3.4d, Scribner Associates, Inc., USA) using appropriate equivalent circuits. The graphical representation of data was carried out using Origin-Pro (version 9.0) software.

### Declaration for the usage of plant materials

We declare that in this experiment, we did not use or not going to use any plants (either cultivated or wild) irrespective of any location. According to our requirements, all the plant-related samples are purchased from the local markets in the U.K. and India, which are completely designated for commercial usage. Experimental research and field study on those samples in this study has complied with the IUCN Policy Statement on Research Involving Species at Risk of Extinction.

## Results and discussions

### Analysis of carbon nanostructure

**Figure 2:**
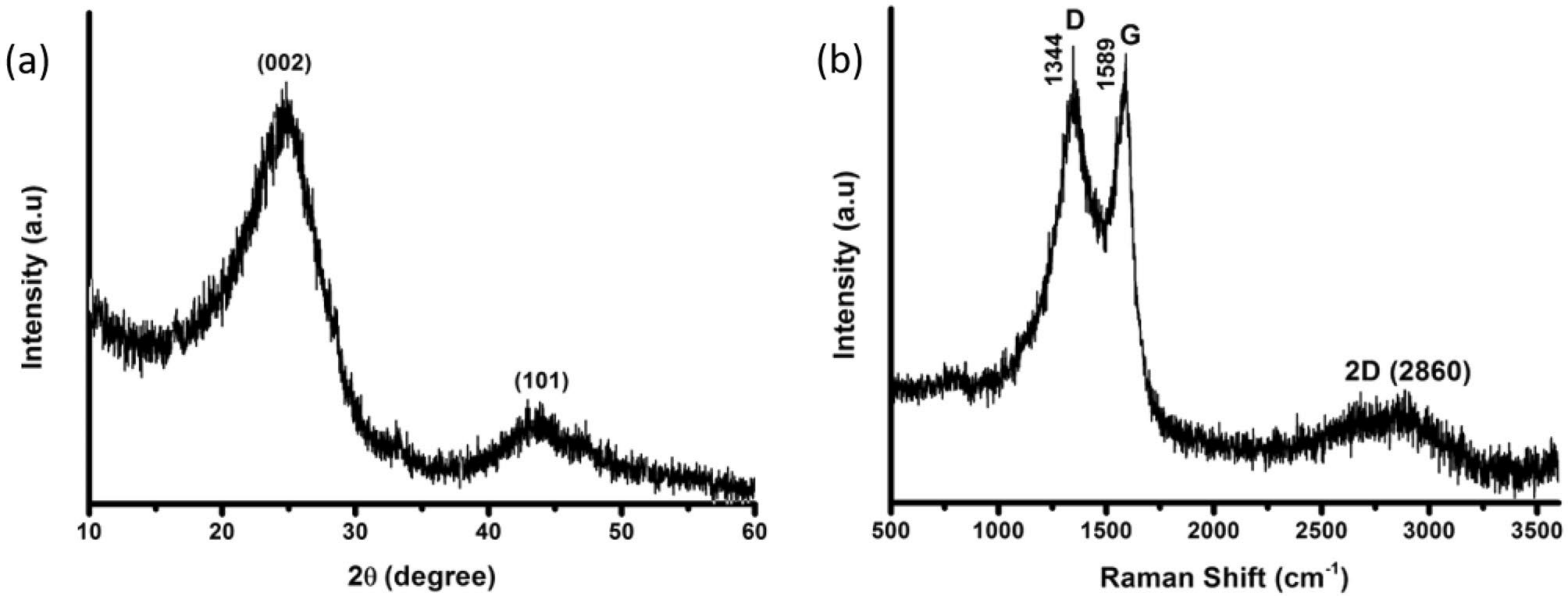
(**a**) XRD pattern of as-synthesized CNP, (**b**) respective Raman spectrum.

**Figure 3:**
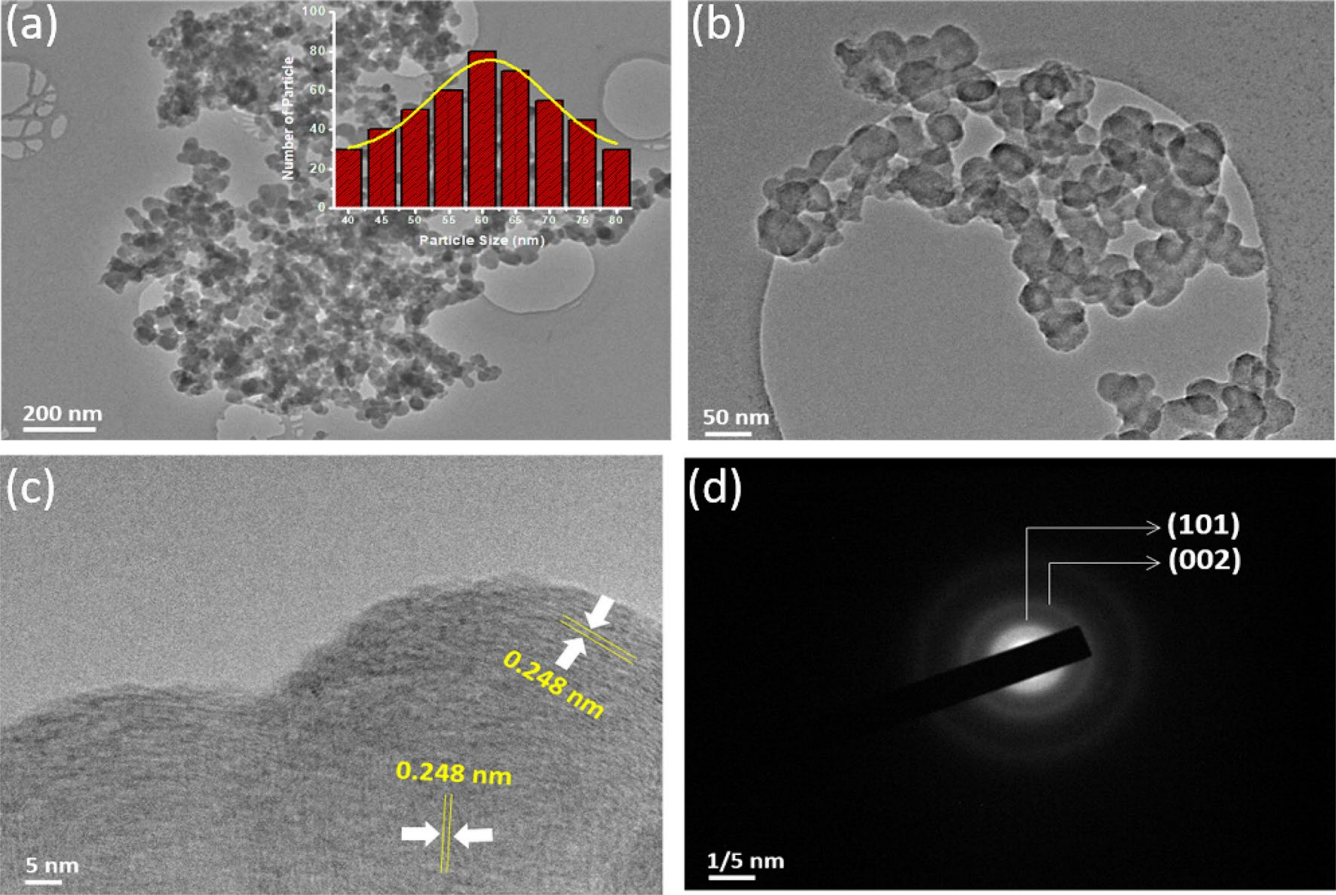
(**a**) and (**b**) TEM bright field images of synthesized CNPs at different magnification (inset: corresponding particle size histogram), respectively, (**c**) HRTEM image of single CNP nanoparticle, (**d**) SAED pattern of CNP.

The carbon nanostructure was synthesized by adopting an ancient method partly with some suitable modifications, as shown in Fig. 1. The detailed process is mentioned in the experimental section. The synthesized CNP were characterized by Raman spectra, XRD (X-ray diffraction), TEM (transmission electron microscopy) and high-resolution TEM (HRTEM) along with selected area electron diffraction (SAED). To begin with, the XRD analysis disclosed that the sample showed broad peaks for (002) at 2θ = 24.47° and (100) at 2θ = 43.32°. In Fig. 2a, the peaks suggest that the sample has C−C (sp^2^) bonding structure with good crystallinity. Raman spectroscopy is one of the most preferred techniques for the examination of carbon materials. So, Raman analysis was carried out to reveal the crystalline nature of the sample. The disordered peak ~ 1344 cm^−1^ (D-band) and graphitic peak ~ 1589 cm^−1^ (G-band), as shown in Fig. 2b, are corresponding to E_2g_ mode, which confirms the graphite related sp^2^-bonding. The *I*_*D*_*/I*_*G*_ ratio of 1.03 indicates a high degree of graphitization^50^. The TEM images of nanoparticles shown in Fig. 3a–d revealed nearly 60 nm (average particle size) carbon nanoparticles are formed. The regular spherical shape of the nanoparticles can be confirmed from the higher magnified TEM image. The SAED pattern of the nanoparticles implies crystalline nature with a layered stacking configuration. HRTEM image manifest d_002_ = 0.248 nm, which is compatible with XRD analysis. Thermogravimetric analysis (TGA) was also conducted for the as-prepared nanoparticles that is given in Fig. S1a, SI. It dictates a high degree of stability upto 600 °C with ~ 3% weight loss, and upto 800 °C, it has fair stability with almost ~ 90% retention of initial weight. From ~ 700 °C the synthesized nanoparticles started oxidizing, and rapid weight loss was observed. The as-prepared CNPs are compared with other previously reported carbon nanoparticles, as shown in Table S1, SI. Among different types, Aloe vera based CNPs and candle soot are highly promising with respect to their conductivity and thermal stability^46,50^. Graphitic CNPs synthesized from the queen of oils (sesame oil) have a good average particle size of ~ 35 nm, although the thermal stability is on the lower side as well as the conductivity, as reported by Das et al^56^. In this study, synthesized CNPs have a relatively larger average particle size of ~ 60 nm, but the conductivity of ~ 8 S cm^−1^ (Screen printed film has a conductivity of ~ 500 S cm^−1^) as shown in Fig. S2, SI and thermal stability are very significant. Again, the presence of a 2D band peak in the Raman spectrum (Fig. 2b) suggests an enhanced graphitic state observed in the as-prepared CNPs. As a whole, the CNPs reported in this work are highly comparable concerning different previously reported CNPs, if not better than them for the application in perovskite solar cells.

### Structural and photovoltaic analysis of HTCE devices

**Figure 4:**
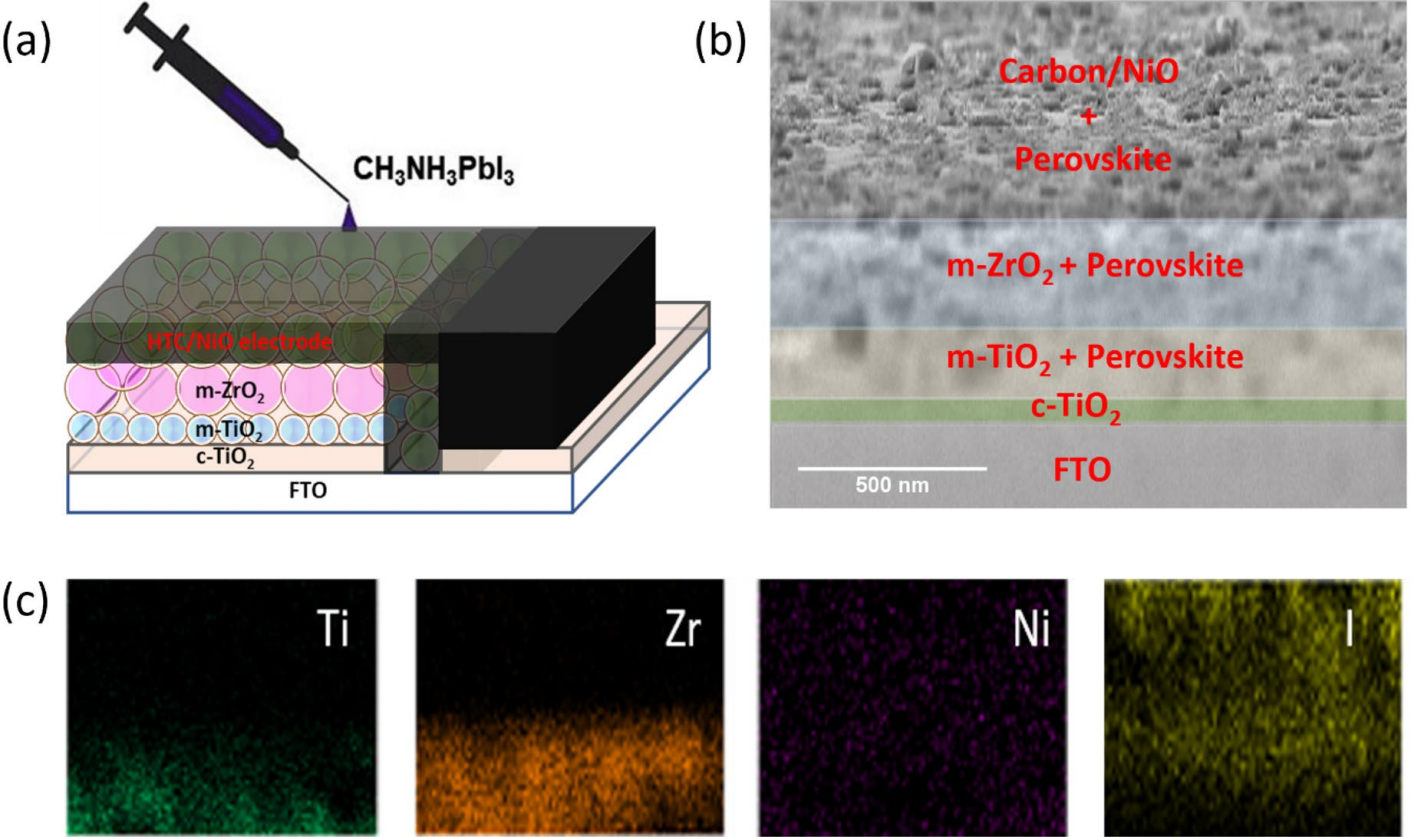
(**a**) Structural representation of HTCN device, (**b**) SEM image of HTCN device showing different layers, (**c**) EDX elemental analysis of Ti, Zr, Ni, I for the HTCN based device.

As this work aims for preparing CNP based high-temperature and low-temperature cathodes, the conductivity test was performed for the HTCN/NiO and LTCN electrodes. The screen printed and blade coated films of HTCN/NiO and LTCN electrodes were found to have a resistance of ~ 6.85 Ω/sq for both cases. Initially, HTCN/NiO based devices were fabricated and characterized using suitable techniques. The Structural representation of the HTCN/NiO device is given in Fig. 4a, which suggests the order of fabrication from top to bottom. Cross-sectional SEM was carried out (Fig. 4b) to confirm the configurational property, which showed the layers' excellent and distinctive nature. The thickness of the m-TiO_2_ layer was ~ 200 nm, and the m-ZrO_2_ layer was ~ 300 nm. The conductive composite electrode had a width of ~ 5 μm in this triple-layered mesoscopic structure. The elemental distribution was observed using EDX (energy-dispersive X-ray spectra) mapping, as shown in Fig. 4c. The excellent distribution of Ti, Zr, Ni and I can be seen from the EDX mapping suggesting prominent deposition of different layers. The distribution of Ni and I indicate the perovskite infiltration through the composite electrode.

Again, the formation of crystalline MAPI was analyzed by XRD separately using the as-prepared precursor solution, which is shown in Fig. S4, SI. The typical peaks at 14.10°, 23.47°, 28.42°, and 30.89° corresponding to the (110), (211), (220), and (213) planes of tetragonal phase of MAPbI_3_, clearly implies the crystalline phase of MAPI.

**Figure 5:**
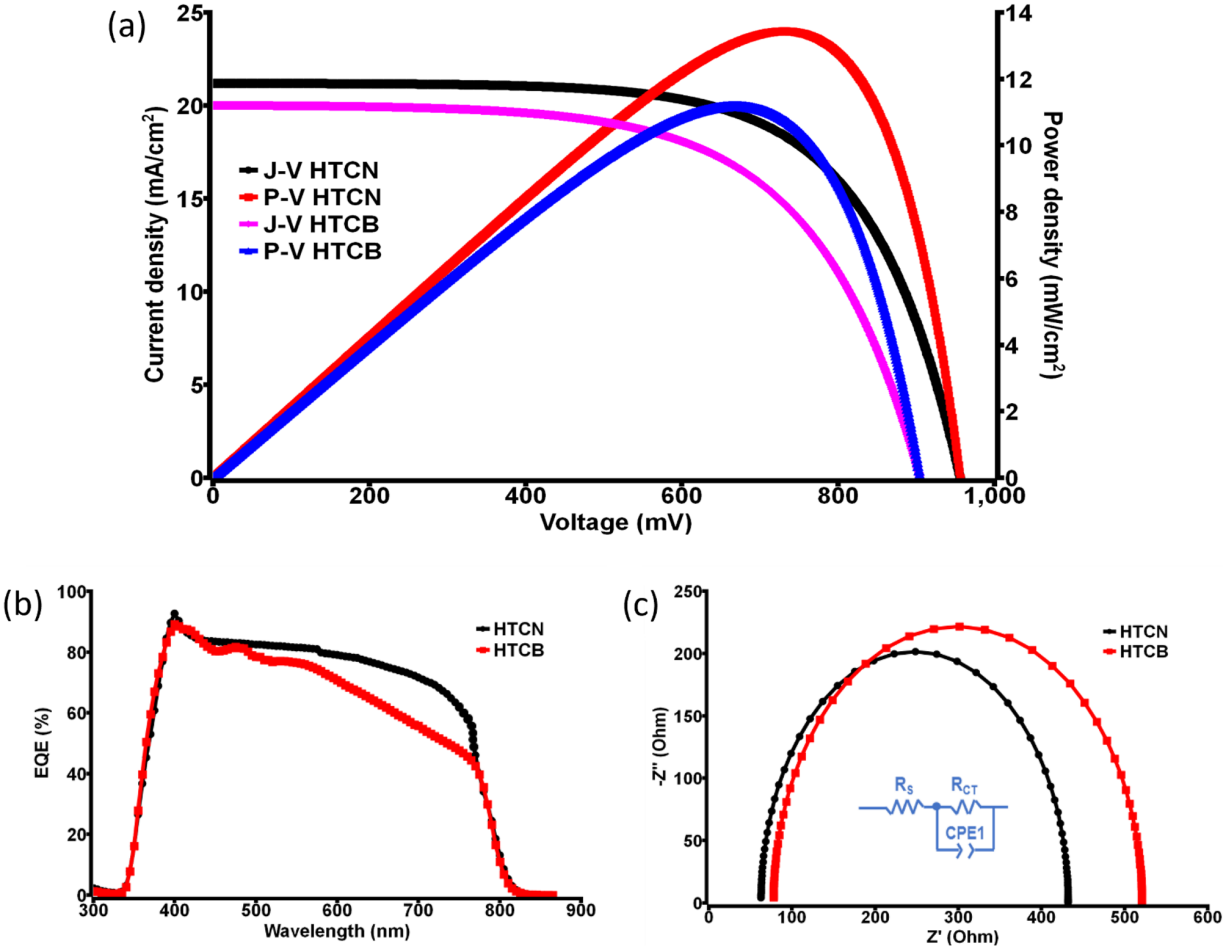
(**a**) J–V and P–V curves of the champion HTCN and HTCB devices, (**b**) EQE of the champion HTCN and HTCB devices, (**c**) EIS representation of the champion HTCN and HTCB devices along with the electrical circuit diagrams.

Performance evaluation of the as-prepared triple-layered mesoscopic devices was initiated by measuring the current versus voltage (*J–V*) characteristics under simulated AM 1.5 (100 mW/cm^2^). For comparison between carbon black and CNP, high-temperature carbon black-based (HTCB) devices were also tested under similar conditions. Photovoltaic performance of the HTCN devices was examined (0.16 cm^2^ active area), and the maximum PCE was found ~ 13.2% having a short-circuit current density (J_SC_) ~ 21.18 mA/cm^2^, open-circuit voltage (V_OC_) ~ 955 mV and FF (fill factor) ~ 0.652, whereas the highest PCE achieved by the HTCB devices was ~ 11.1% as shown in Fig. 5a. It implies that the synthesized carbon nanoparticles are much more suitable than the commercial carbon black. A detailed tally of the performance of champion devices for both cases is given in Table 1. Shunt resistance (R_sh_) of the devices are calculated from the typical behavior of J–V characteristic under ideal one-sun illumination condition of a solar cell using the Shockley equation, where J_SC_ can be written as Eq. (1)^57^.

1 $${J}_{sc}={J}_{Ph}-{J}_{0}\left[\mathrm{exp}\left(\frac{q\left(V-J{R}_{s}\right)}{nkT}\right)-1\right]-\frac{V-J{R}_{s}}{{R}_{sh}}$$

where J_Ph_—photocurrent density, J_0_—saturation current density at reverse bias, q—elementary charge, R_s_—and R_sh_—the series and shunt resistances, respectively, n—the diode ideality factor, k—Boltzmann’s constant (1.38 × 10^−23^ JK^−1^), T—room temperature (298 K).

**Table 1:** Photovoltaic measurement parameters of champion HTCN and HTCB devices (active area 0.16 cm^2^).

Device type	J_SC_ (mA/cm^2^)	V_OC_ (mV)	FF (%)	PCE (%)	Power density (mW/cm^2^)	R_S_ (Ohm)	R_sh_ × 10^3^ (Ohm)
HTCN	21.18 ± 0.2	955.0 ± 1.5	65.2 ± 1	13.2 ± 0.3	13.2 ± 0.3	62.8 ± 1.5	42.0 ± 8
HTCB	20.0 ± 0.2	904.8 ± 2	61.4 ± 1	11.1 ± 0.3	11.1 ± 0.3	77.71 ± 2	14.0 ± 6

To confirm the better efficacy of synthesized CNT, the external quantum efficiency (EQE) of the devices were measured, which exhibited a broad peak over the range of 300–800 nm with a maximum value of ~ 90% for both the HTCN and HTCB devices, including the effects of optical losses originated from transmission and reflection. Although from Fig. 5b, one can clearly understand that the EQE versus wavelength is more prominent for HTCN device relative to the HTCB device. That is why the calculated value of the integrated photocurrent density for the champion HTCN device is found to be 19.59 mA/cm^2^, and for the champion HTCB device is 17.4 mA/cm^2^. Thus, the values of average integrated photocurrent densities of different carbon electrode-based PSCs closely matches with current densities obtained from the *J–V* curve.

Further, an understanding of internal electrical properties at different interfaces of the HTCN perovskite devices was made by electrochemical impedance spectroscopy (EIS) measurements. The Nyquist plot with the equivalent circuit diagram of the concerned PSCs was monitored under dark at 0.8 V bias from 1 MHz to 10 mHz, as shown in Fig. 5c. In the circuit diagram (inset of Fig. 5c for HTCN device), R_s_ represents the series resistance, including the FTO and carbon counter electrode resistance. Generally, the charge transfer resistance (R_CT_) functions at the perovskite/carbon interface, and the charge recombination resistance (R_rec_) operates at TiO_2_/MAPbI_3_ interface. A relatively small value of R_CT_ for the HTCN device implies better charge collection ability at CNT/NiO and MAPI interface relative to the HTCB device. The R_s_ value of the HTCN device was found to be smaller than that of the commercial carbon-based device, as mentioned in Table 1. It resembles the fact that PCE should be higher if the R_s_ value is on the lower side. Data reliability plays a crucial role in understanding solar cells for which box and whiskers plots related to the J–V performance of the ten best HTCN devices are given in the SI, Fig. S5.

### Structural and photovoltaic analysis of LTCE devices

**Figure 6:**
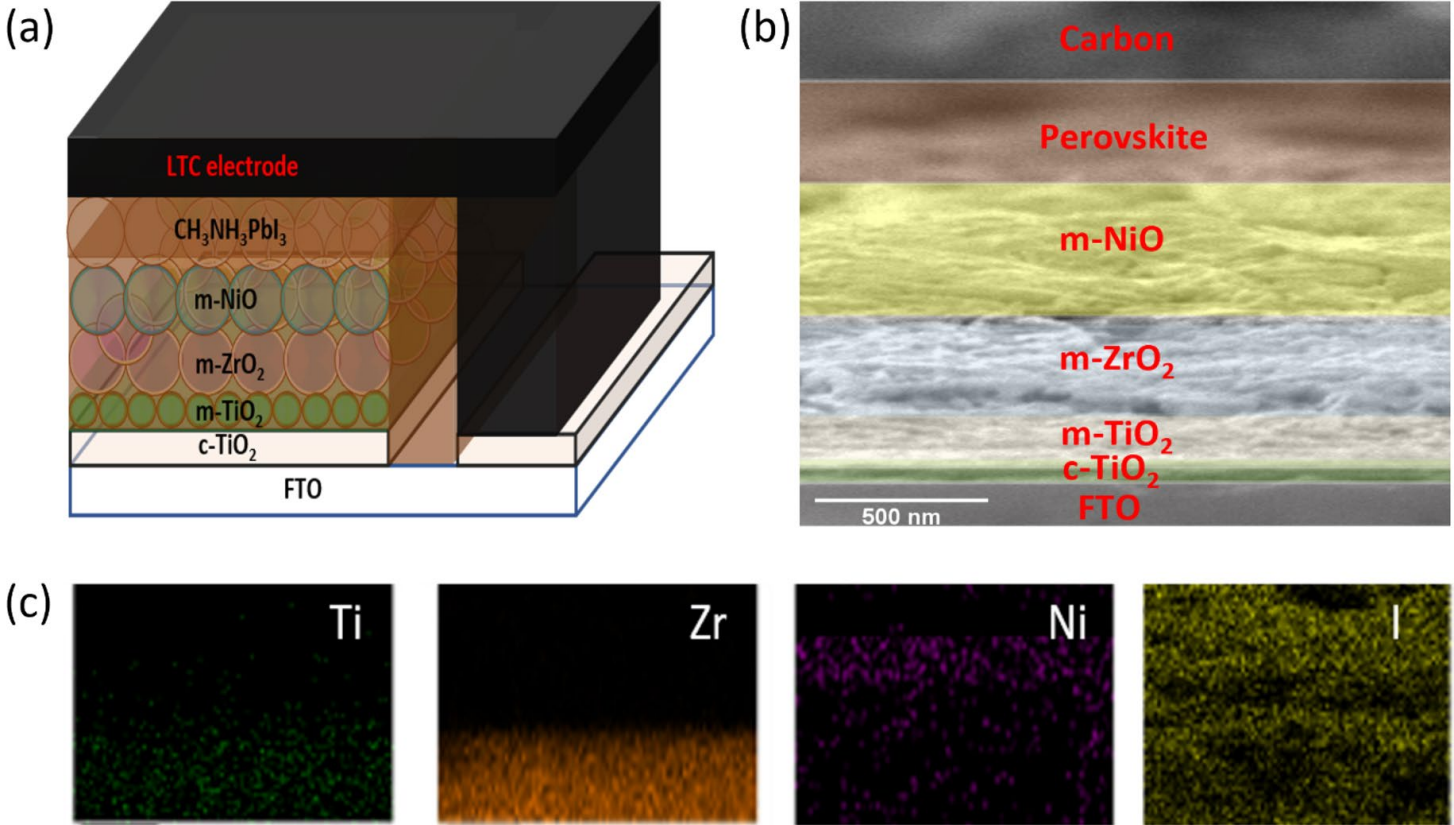
(**a**) Structural representation of LTCN device, (**b**) SEM image of LTCN device showing different layers, (**c**) EDX elemental analysis of Ti, Zr, Ni, I for the LTCN based device.

The LTCE devices were examined with the help of conformational and performance analysis. In Fig. 6a, the overall structure of different layers deposited for the LTCE devices is shown. The formation of separate films of mesoporous-NiO and MAPI is the primary difference of LTCE devices with HTCE devices. The cross-sectional SEM image in Fig. 6b unveils the structural dissimilarities displaying separate c-TiO_2_, m-TiO_2_, m-ZrO_2_, m-NiO, perovskite and electrode layers. The analysis suggested that the m-TiO_2_, m-ZrO_2_, m-NiO, perovskite layers have a thickness of ~ 200 nm, ~ 300 nm, ~ 400 nm and ~ 300 nm, respectively. EDX examination also showed (Fig. 6c) the precise elemental analysis of Ti, Zr, Ni, and I, proving excellent deposition of different materials. Mapping of Ni and I suggest perovskite deposition on top of the mesoporous NiO layer.

**Figure 7:**
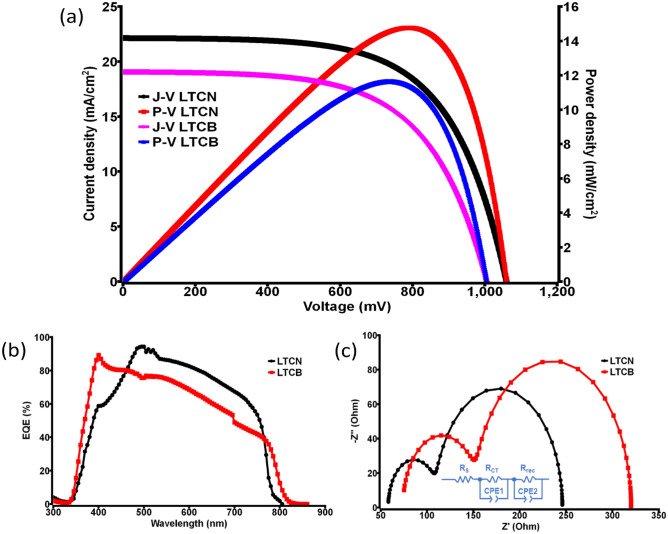
(**a**) J–V and P–V curves of the champion LTCN and LTCB devices, (**b**) EQE of the champion LTCN and LTCB devices, (**c**) EIS representation of the champion LTCN and LTCB devices along with the electrical circuit diagrams.

The impressive structural properties have to produce a quality performance as a solar cell. To find out the performance of LTCN devices, the J–V curve was analyzed in the first place using simulated AM 1.5 (100 mW/cm^2^). Similar to HTCN based devices, the LTCN devices were also compared with low-temperature carbon black (LTCB) based counter electrode. The photovoltaic performance of LTCN devices was able to achieve a standout PCE of ~ 14.2% along with J_SC_ of 22.12 mA/cm^2^, V_OC_ of 1060.2 mV and FF of 60.5%, whereas the highest PCE achieved by the LTCB devices was ~ 11.3%, as shown in Fig. 7a and Table 2. The effectiveness of CNP is way ahead of the carbon black in terms of PCE, which proves the significance of the as-synthesized CNP. J–V performance variations of 10 LTCN devices are given in the box and whiskers plot in SI, Fig. S5 to display PCE, J_SC_, V_OC_, and FF variations.

**Table 2:** Photovoltaic measurement parameters of champion LTCN and LTCB devices (active area 0.16 cm^2^).

Device type	J_SC_ (mA/cm^2^)	V_OC_ (mV)	FF (%)	PCE (%)	Power density (mW/cm^2^)	R_s_ (Ohm)	R_sh_ × 10^3^ (Ohm)
LTCN	22.12 ± 0.2	1060.2 ± 2	60.5 ± 1.5	14.2 ± 0.35	14.1 ± 0.35	58.16 ± 2	50.0 ± 5
LTCB	19.1 ± 0.2	1005.0 ± 2.5	59.0 ± 1	11.3 ± 0.35	11.3 ± 0.35	73.92 ± 1.5	15.0 ± 5

After PCE measurements, using the IPCE (incident photon to electron conversion) equipment, the EQE was tested, reflecting a maximum of ~ 90% near 500 nm for the LTCN device. From the overlap integral of the IPCE spectra shown in Fig. 7b, the integrated photocurrent density of the champion LTCN device was calculated and found to be 19.0 mA/cm^2^, whereas LTCB achieved ~ 16.8%, which closely matches with their J_SC_ acquired from J–V characterization. Additionally, The internal electrical property was determined using EIS measurement similar to HTCE devices. The Nyquist plot shown in Fig. 7c suggests the series resistance R_s_ is lower in the champion LTCN device (58.16 Ω) relative to the LTCB device (73.92 Ω). Also, the charge transfer resistance (R_CT_) between the carbon/perovskite interface was found to be smaller for the LTCN based devices suggesting better charge collection in the device. Thus, the CNP based devices are much more promising in all aspects in comparison to the carbon black based devices.

### Comparative analysis between HTCE and LTCE photovoltaic devices

**Figure 8:**
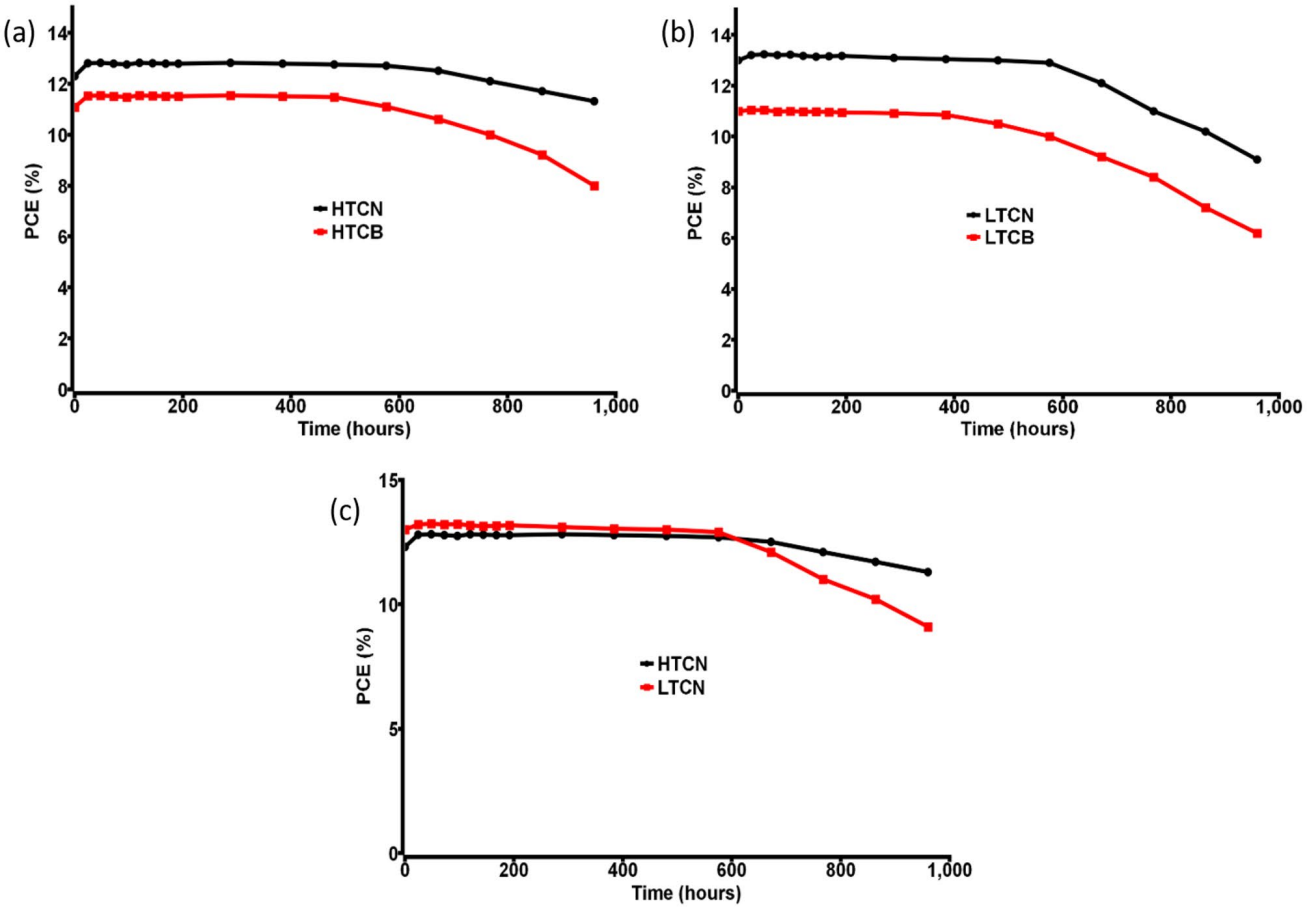
(**a**) Stability comparison between HTCN and HTCB devices, (**b**) stability comparison between LTCN and LTCB devices, (**c**) stability comparison between HTCN and LTCN devices.

The utilization of NiO as HTM for both the HTCE and LTCE based devices dramatically influences the efficacy, and that is well reported in previous research works^58^. For comparison, we prepared few devices without NiO and found a maximum PCE of ~ 10%. Performance variation of carbon black and CNP based devices with NiO are primarily due to the large surface area of the CNPs, high degree of graphitization and amplified conductivity, which in turn enhances the efficacy of the CNP based devices influencing the charge transport property. The observed stability (ambient conditions) of both the unencapsulated HTCN and LTCN based devices are much higher than their respective competitors having carbon black electrodes. The stability of perovskite is always an issue, although device configuration and materials have a critical role in increasing or decreasing the stability even further. We believe the outcome of the stability test shown in Fig. 8 is largely due to the use of CNP as the counter electrode. The small porosity of the CNP based electrode plays a significant role in obstructing the permeation of moisture and excess oxygen through the device, which increases the lifetime of the CNP devices compared to carbon black devices.

The most exciting factor of this study is the comparison between the HTCN and LTCN devices. From materials preparation and fabrication time to apparent cost of production and performance analysis, these two processes have some advantages over one another. Considering the experimental process, the benefits of the high-temperature process with respect to the low-temperature process are reduced time of sample preparation due to composite electrode, reduced time of fabrication process, as well as minimizing the cost of production by using a small quantity of starting materials. The sequential layer deposition for HTCN devices was performed in 6 steps, whereas LTCN devices needed 7 steps. Adding to that separate hole transport layer for LTCN devices makes them a little costlier. As a solar cell, performance plays the most significant role in deciding its fate. Although the PCE of LTCN devices was found to be higher than that of the HTCN devices, the FF of the LTCN devices was much smaller than the HTCN devices. The reason behind this low FF of LTCN devices can be explained by the comparatively small R_rec_ values observed from the EIS. The high charge recombination rate due to the small R_rec_ value of the LTCN devices reduce their FF. Again the small values of charge exchange resistance R_CT_ enhance the charge collection ability of counter electrode and perovskite in LTCN devices, effectively increasing their current and voltage. The most suitable logical explanation behind increased charge recombination is the presence of a separate NiO layer in LTCN devices^28^. Moving forward to the stability comparison between the LTCN and HTCN devices, It was found that LTCN devices rapidly lost their PCE after 600 h, whereas HTCN devices had very high stability retaining almost 90% of their PCE after 1000 h (Fig. 8c). The possible reason behind this phenomena can be the small porosity of the NiO/CNT composite electrode in the HTCN device, which reduces the penetrating power of the moisture/oxygen^45^. Again, the presence of a separate perovskite layer makes the low temperature based devices more prone to degradation as the halide perovskite can quickly come in contact with the moisture/oxygen^39^. From the entire comparison, it is quite clear that in ambient conditions, high-temperature carbon nanoparticle-based devices are most effective considering different factors, although they have lesser PCE. However, the stability of both the devices can be increased significantly (> 1000 h) by encapsulating them, and in that case, due to higher PCE, low-temperature carbon nanoparticle-based devices have the edge over their competitors. As a whole, the HTCN and LTCN devices are highly promising in their performance. If PCE is the main criteria, then one can look for the LTCN devices, but if stability with PCE is the major criteria, it is needless to say that HTCN devices are much more promising. These results need further improvement for PSC to be representative of the desired decadal operational lifetime. In the meantime, these reports provide a useful metric to help the perovskite community to increase reliability.

Parallel to their progressive development, PSCs have shown excellent potential for more environmentally sustainable systems. Furthermore, we believe every new configured PSCs requires a complete life cycle analysis (LCA) to understand their environmental impact in relation to global marketing. Various works are going on for LCA studies related to perovskite photovoltaics^59^. Usually, several parameters have to be considered like primary energy consumption, the carbon footprint, the performance ratio, component production, module manufacturing, the module usage, insolation, lifetime and recycling for a proper understanding of the LCA of c-PSCs in order to determine the cost of the photovoltaic technology^60,61^. Research related to the LCA study, detailed cost estimation and environmental influence with c-PSC is urgently required, and we believe our proposed photovoltaic solution can be highly beneficial in this purpose.

## Conclusion

In summary, we developed a novel and cost-effective synthesis process of CNP counter electrode/HTM for PSCs that is time-saving and provides high air stability compared to conventional techniques. The morphological study of the materials and devices and the fabrication pathway offers remarkable performance for the PSCs. This printable CNP plays a vital role in the air-stable and moisture-stable mesoscopic perovskites. The stability test of HTCN PSCs showed ~ 1000 h air stability with negligible efficiency loss having a maximum PCE of 13.2%. On the other hand, the LTCN PSCs showed 14.2% PCE with ~ 650 h air stability with minor efficiency loss during the stability study. Although the developed CNP based PSCs showed moderate PCE, it has high stability in ambient conditions with almost zero commercial value, which makes it one of the cheapest PSC toward commercialization. This novel mustard oil assisted cotton-soot based carbon counter electrode/hole-conductor with a unique synthesis method, and printing feasibility allows long life to the MAPbI_3_-absorbing layer, resulting in retarded deterioration of the perovskite layer. The comparison between low-temperature non-composite and high-temperature composite HTL/counter electrode-based devices expands the possibility of carbon-based PSCs. It gives the idea of an advantageous fabrication method when someone considers carbon-based perovskite solar cells. We firmly believe that this study can be beneficial to increase the performance of different mixed-halide perovskites to > 20% PCE using carbon-based materials. For large-scale terrestrial deployment of perovskites, maintaining these high efficiencies while achieving stability and scaling will be necessary. In this regard, the synthesis approach of preparing carbon electrodes will be highly encouraging.

## Supplementary Information

Supplementary Information.

## Data Availability

The data that support the plots within this paper and other findings of this study are available from the corresponding author upon reasonable request.
