# Supplementary material for: Cotton soot derived carbon nanoparticles for NiO supported processing temperature tuned ambient perovskite solar cells
Source: Sci Rep. 2021 Dec 3;11:23388. doi: 10.1038/s41598-021-02796-w (PMC8642405; doi:10.1038/s41598-021-02796-w)
Supplement: Supplementary file 1 — Supplementary Information. [file 41598_2021_2796_MOESM1_ESM.docx]

**Supporting Information**

**Cotton soot derived carbon nanoparticles for NiO supported processing temperature tuned ambient perovskite solar cells**

Shubhranshu Bhandari,*^a^ Anurag Roy,^a^ Mir Sahidul Ali,^b^ Tapas Kumar Mallick^a^, and Senthilarasu Sundaram*^a^

^a^Environment and Sustainability Institute (ESI), Penryn Campus, University of Exeter, Cornwall, TR10 9FE, U.K.

^b^Department of Polymer Science and Technology, University of Calcutta, 92 A.P.C Road, Kolkata, 700009, West Bengal, India

* Corresponding authors

E-mail: [sb964@exeter.ac.uk](mailto:sb964@exeter.ac.uk), [s.sundaram@exeter.ac.uk](mailto:s.sundaram@exeter.ac.uk)

**Supplementary Note 1:** Thermogravimetric analysis of as-prepared carbon nanoparticles (CNP) shown in Fig S1a suggests high thermal stability of nanoparticles. Only ~6% weight loss was found during TGA analysis at 800 °C, which indicate suitability for high-temperature treatment.


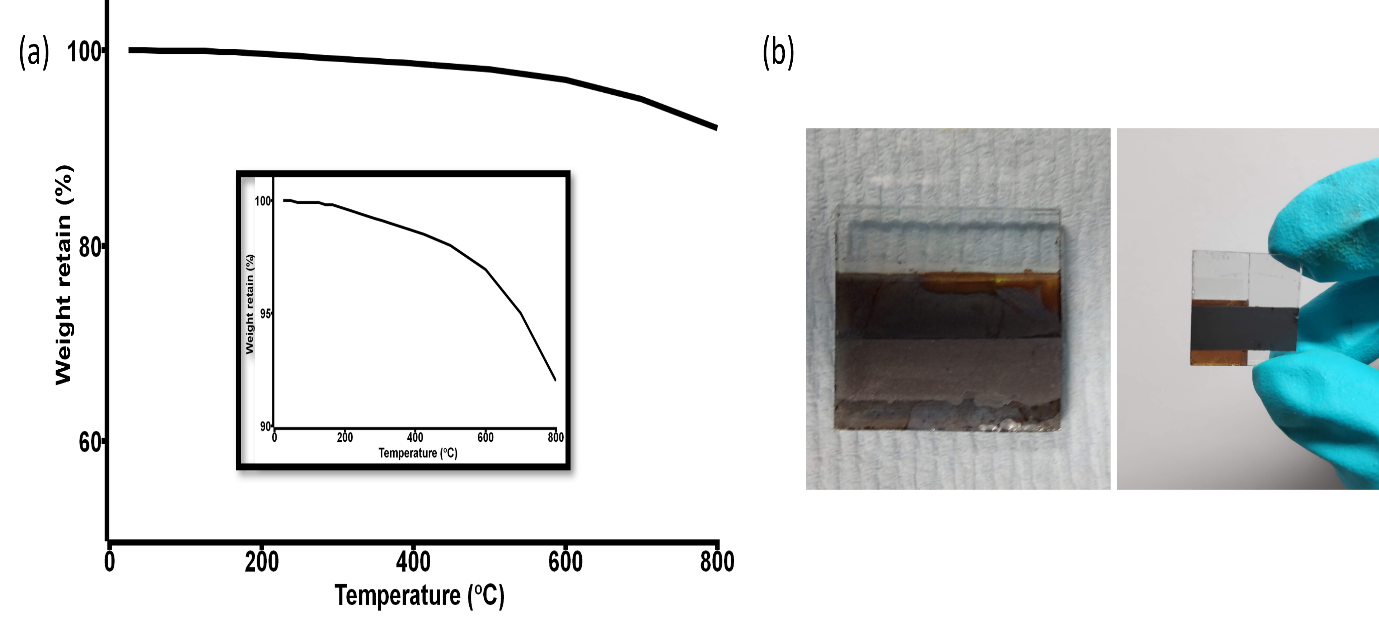


**Fig. S1** (a) Thermogravimetric analysis (TGA) curves of as-synthesized CNP powder sample at 800 °C, (b) Image of as prepared HTCN and LTCN based PSCs.

**Supplementary Note 2:** The conductivity of as-prepared CNTs placed on a petri-dish are measured at different positions, which shows an average value of ~8 S.cm^-1^. Again the resistance and conductivity measurement of CNP electrode (screen printed and blade coated layer) for both high-temperature and low-temperature purposes were tested and fixed at ~ 6.85 Ω/sq and ~475 S.cm^-1^, respectively.

**
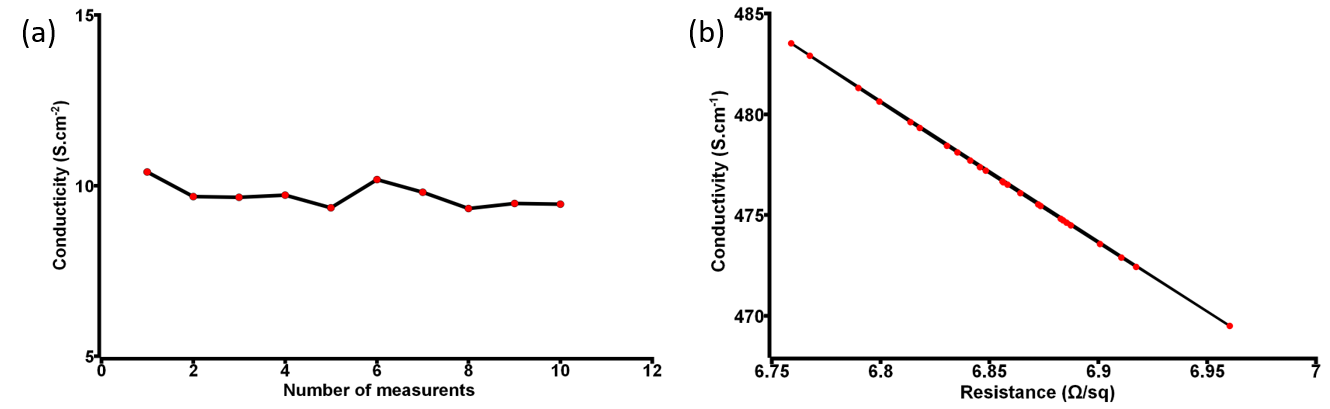
**

**Fig. S2** (a) Conductivity of synthesized CNPs at different points placed on a petri-dish, (b) Sheet resistance vs. conductivity measurement of coated CNP paste using ‘Osilla 4-point probe instrument’.

| **Table S1 Comparison between different carbon nanoparticles** | | | | | |
| --- | --- | --- | --- | --- | --- |
| **Sample** | **Particle size (nm)** | **TGA analysis** | **Conductivity (S.cm^-1^)** | **Resistance (Ω)** | **Reference** |
| Fluorescent carbon nanoparticles | 2-6 | NA (not available) | NA | NA | ^1^ |
| Candle soot | 30 | NA | ~5 | NA | ^2^ |
| graphitic carbon nanoparticles from queen of oils | 30-40 | Stable upto ~600 °C | NA | ~0.3 | ^3^ |
| Corn stalk soot | 6-18 | NA | NA | NA | ^4^ |
| Aloe vera based CNP | 45 | Stable upto 1000 °C | NA | 6.99 Ω/sq  For screen printed film | ^5^ |
| This work (cotton soot) | 60 | Stable upto 800 °C | ~8 for CNP and ~500 for screen printed film | 6.85 Ω/sq  For screen printed film |  |


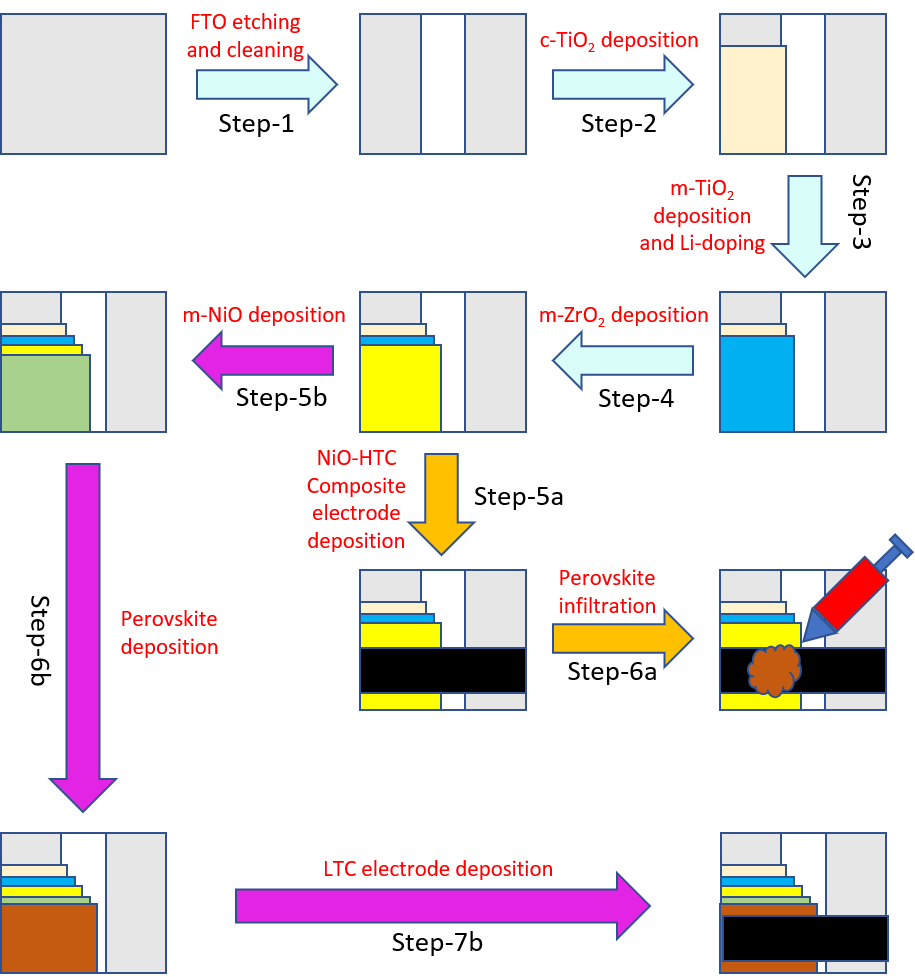


**Fig. S3** Stepwise fabrication process of HTCE and LTCE devices starting from FTO etching and finishing with perovskite infiltration and electrode deposition for the HTCN and LTCN based devices, respectively.

**Supplementary Note 3:**  The XRD pattern of synthesized CH_3_NH_3_PbI_3_ thin films on the FTO glass substrate is shown in Fig S3. Except for the signals of FTO glass and anatase TiO_2_ shown with yellow and red, respectively, all remaining signals are responsible for MAPbI_3_ perovskite. The typical peaks at 14.10°, 23.47°, 28.42°, and 30.89° correspond to the (110), (211), (220), and (213) planes of tetragonal phase of MAPbI_3_, respectively. XRD study confirms the phase purity and crystalline features of MAPbI_3_.


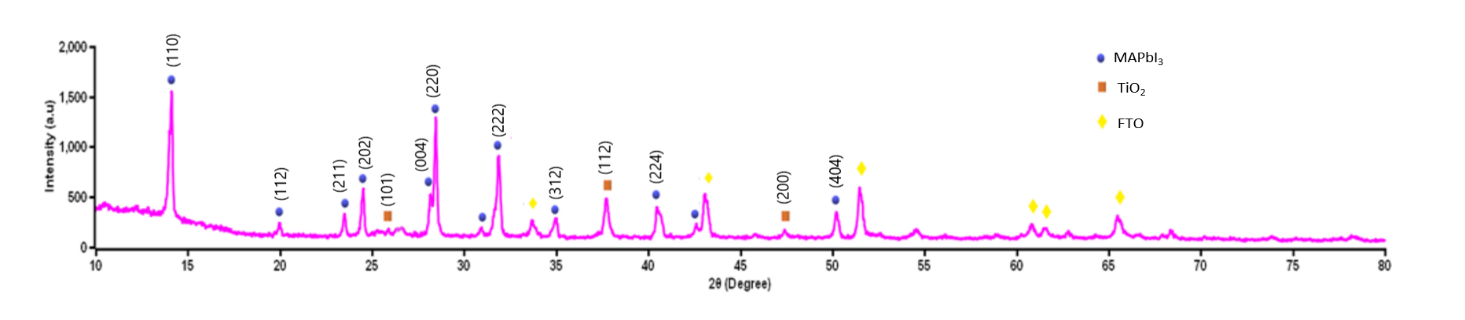


**Fig. S4** XRD patterns of the MAPbI_3_/TiO_2_/FTO device (in blue) with major peaks for (110), (211), (220), and (213) planes for MAPbI_3_.

**Supplementary Note 4:** The J-V characteristics of HTCN and LTCN devices were observed to get an idea about their performances. Fig S5 gives the variance of V_OC_, J_SC_, fill factor and PCE values for a batch of 10 devices for both types of devices. The PCE values range from 12.8% to 10.6% for the HTCN based devices and 13.2% to11.1% for the LTCN based devices.


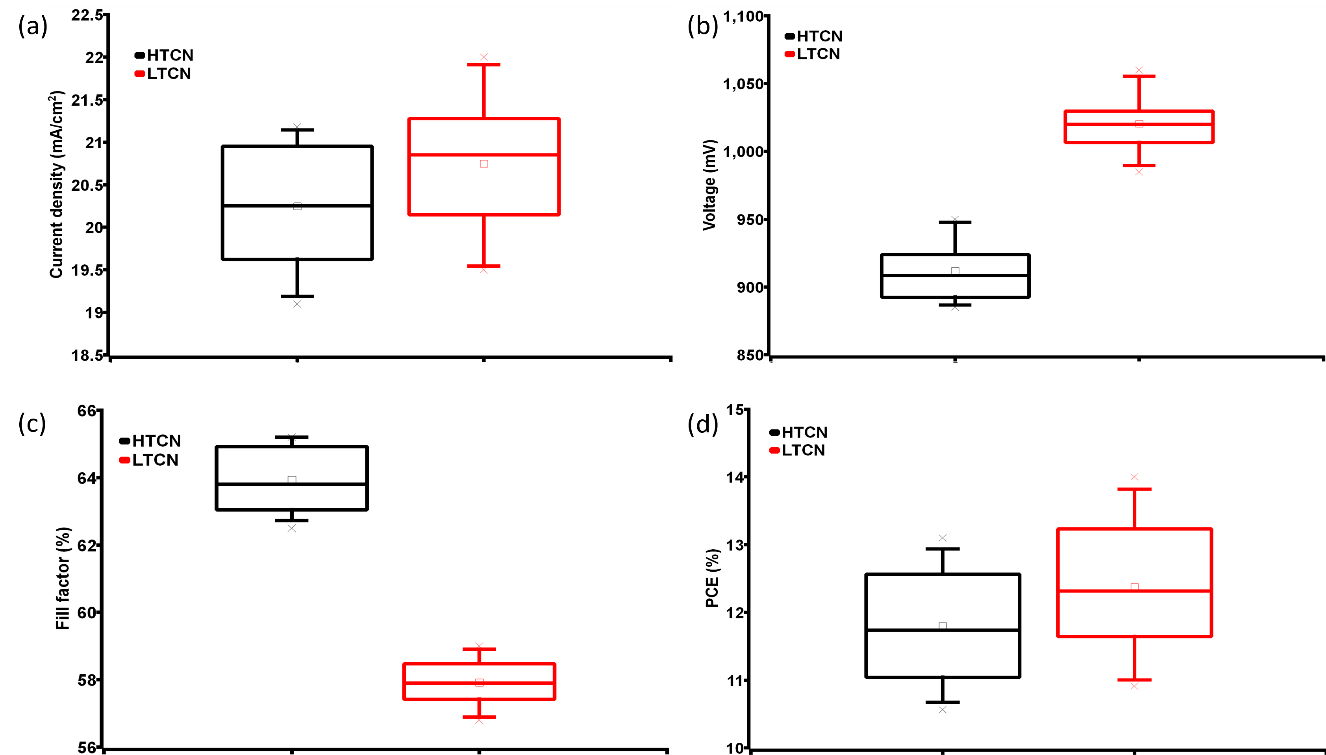


**Fig. S5** Box and whiskers plot of current density, voltage, FF and PCE for each HTCN (10 devices) and LTCN (10 devices) based devices.

**References**

1. Ray, S. C., Saha, A., Jana, N. R. & Sarkar, R. Fluorescent Carbon Nanoparticles: Synthesis, Characterization, and Bioimaging Application. *J. Phys. Chem. C* **113**, 18546–18551 (2009).

2. Wei, Z. *et al.* Cost-efficient clamping solar cells using candle soot for hole extraction from ambipolar perovskites. *Energy Environ. Sci.* **7**, 3326–3333 (2014).

3. Das, P. P. *et al.* Multiband Fluorescent Graphitic Carbon Nanoparticles from Queen of Oils. *ACS Sustain. Chem. Eng.* **6**, 10127–10139 (2018).

4. Li, Y., Chen, T. & Ma, Y. Nanosized carbon dots from organic matter and biomass. *J. Wuhan Univ. Technol. Sci. Ed.* **31**, 823–826 (2016).

5. Mali, S. S., Kim, H., Patil, J. V & Hong, C. K. Bio-inspired Carbon Hole Transporting Layer Derived from Aloe Vera Plant for Cost-Effective Fully Printable Mesoscopic Carbon Perovskite Solar Cells. *ACS Appl. Mater. Interfaces* **10**, 31280–31290 (2018).
